# Myosin-II proteins are involved in the growth, morphogenesis, and virulence of the human pathogenic fungus *Mucor circinelloides*

**DOI:** 10.3389/fcimb.2022.1031463

**Published:** 2022-12-16

**Authors:** Trung Anh Trieu, Phuong Anh Nguyen, Mai Ngoc Le, Huy Nhat Chu

**Affiliations:** ^1^ Department of Genetics - Biochemistry, Faculty of Biology, Hanoi National University of Education, Hanoi, Vietnam; ^2^ Environmental Bioremediation Laboratory, Institute of Biotechnology, Vietnam Academy of Science and Technology, Hanoi, Vietnam; ^3^ Graduate University of Science and Technology, Vietnam Academy of Science and Technology, Hanoi, Vietnam

**Keywords:** dimorphism, fungal infection, mucormycosis, myosin, pathogenesis

## Abstract

Mucormycosis is an emerging lethal invasive fungal infection. The infection caused by fungi belonging to the order Mucorales has been reported recently as one of the most common fungal infections among COVID-19 patients. The lack of understanding of pathogens, particularly at the molecular level, is one of the reasons for the difficulties in the management of the infection. Myosin is a diverse superfamily of actin-based motor proteins that have various cellular roles. Four families of myosin motors have been found in filamentous fungi, including myosin I, II, V, and fungus-specific chitin synthase with myosin motor domains. Our previous study on *Mucor circinelloides*, a common pathogen of mucormycosis, showed that the Myo5 protein (ID 51513) belonging to the myosin type V family had a critical impact on the growth and virulence of this fungus. In this study, to investigate the roles of myosin II proteins in *M. circinelloides*, silencing phenotypes and null mutants corresponding to myosin II encoding genes, designated *mcmyo2A* (ID 149958) and *mcmyo2B* (ID 136314), respectively, were generated. Those mutant strains featured a significantly reduced growth rate and impaired sporulation in comparison with the wild-type strain. Notably, the disruption of *mcmyo2A* led to an almost complete lack of sporulation. Both mutant strains displayed abnormally short, septate, and inflated hyphae with the presence of yeast-like cells and an unusual accumulation of pigment-filled vesicles. *In vivo* virulence assays of myosin-II mutant strains performed in the invertebrate model *Galleria mellonella* indicated that the *mcmyo2A*-knockout strain was avirulent, while the pathogenesis of the *mcmyo2B* null mutant was unaltered despite the low growth rate and impaired sporulation. The findings provide suggestions for critical contributions of the myosin II proteins to the polarity growth, septation, morphology, pigment transportation, and pathogenesis of *M. circinelloides.* The findings also implicate the myosin family as a potential target for future therapy to treat mucormycosis.

## Introduction

Mucormycosis (previously termed zygomycosis) is a potentially fatal invasive fungal infection caused by species belonging to the order Mucorales. Mucormycosis occurs primarily in immunosuppressed patients. However, immunocompetent patients who suffered severe injuries that primarily break the integrity of their physical barriers (skin or mucosa) can also be affected. The disease is characterized by an accelerated development with thrombosis, tissue infarction, and subsequent necrosis due to the destructive invasion into blood vessels and other vital organs (Chakrabarti, 2020; Skiada et al., 2020). Successful management of mucormycosis requires early diagnosis, reversal of underlying predisposing factors, surgical debridement, and prompt administration of adequate antifungal agents. However, given the difficulties in diagnosis and treatment, properly controlling mucormycosis is still challenging (Katragkou et al., 2014; Skiada et al., 2018). The mortality of this infection remains unacceptable, generally ranging from 31% to 68% and might be up to 96%, depending on underlying risk factors, site of infection, pathogen type, and treatment (Jeong et al., 2019; Hassan and Voigt, 2019). Although considered a rare infection, the prevalence of mucormycosis is increasing globally, varying from 0.005 to 1.7 per million population. This figure is 80 times higher (0.14 per 1000 people) in India (Skiada et al., 2020). Notably, the outbreak of the coronavirus disease 2019 (COVID-19) has led to a burdensome upsurge of mucormycosis. Cases of COVID-19-associated mucormycosis (CAM) have been increasingly recognized, particularly in India, where more than 47,000 cases of mucormycosis were reported in only three months (May to July 2021) during the second wave of the pandemic (Muthu et al., 2021). To effectively control this emergent infection, further understanding of the pathogenesis and molecular mechanism of mucormycosis and CAM is urgently necessary.

Pathogen virulence factors play a key role in the invasion and damage process. Several critical factors previously described in other fungi are also directly involved with the virulence of Mucorales causing mucormycosis. These include the high-affinity iron uptake system (Ibrahim et al., 2010; Liu et al., 2015; Navarro-Mendoza et al., 2018), mechanism of dimorphism (Li et al., 2011; Lee et al., 2013), and azole resistance (Vitale et al., 2012; Dannaoui, 2017). Additionally, recent advances in novel molecular and genetic tools have enabled the characterization of new genes, pathways, and molecular mechanisms controlling the pathogenic potential of Mucorales and their interactions with the host (Calo et al., 2014; Gebremariam et al., 2014; Lee et al., 2015; Patiño-Medina et al., 2017; López-Fernández et al., 2018; Patiño-Medina et al., 2019; Patiño-Medina et al., 2020; Vellanki et al., 2020). Particularly, the discovery and utilization of RNA interference (RNAi) mechanism in *Mucor circinelloides*, a classic model to study mucormycosis pathogen, has allowed rapid screening and identification of virulence factors (Nicolás et al., 2003; Garre et al., 2014; Ruiz-Vázquez et al., 2015). With this approach, our previous study resulted in the discovery of two factors that are critically required for the proliferation and, hence, the effective infection of the fungus; one of them was *mcmyo5* encoding a crucial transporter of the myosin V family (Trieu et al., 2017).

Myosin is a diverse superfamily of actin-based motor proteins that play various cellular functions (Coluccio, 2020). Myosin proteins are typically constructed of three functional subdomains: (1) the N-terminal head with the motor domain which is an ATP-binding region that interacts with actin, (2) the neck domain which binds light chains or calmodulin, and (3) the C-terminal tail region, which serves to anchor and position the motor domain so that it can interact with actin (Sweeney et al., 2020). Four classes of myosin have been found in filamentous fungi. These include myosin I, myosin II, myosin V, and fungus-specific chitin synthase with myosin motor domains. Single-headed class I myosin motors have been implicated in multiple cellular functions including control of plasma membrane tension, tethering of membrane proteins, endocytosis, exocytosis, and cell–cell interaction (McIntosh and Ostap, 2016). Studies on common pathogenic fungi, including *Candida albicans, Aspergillus nidulans, Neurospora crassa*, and *Fusarium graminearum*, have shown that myosin I proteins are essential for the development of hyphae, polarized growth, secretion, and toxisome formation (McGoldrick et al., 1995; Oberholzer et al., 2002; Fernando et al., 2016; Guangfei et al., 2018). Class II myosin, the conventional two-headed myosin protein first identified in muscle, is the primary motor protein responsible for cytokinesis in non-muscle cells through involvement in the cytokinetic actomyosin contractile ring (Maciver, 1996; Pollard, 2010). In fungi, the involvement of myosin II members in cytokinesis has been described in both budding and fission yeasts (Xiang and Oakley, 2010). In filamentous fungi, type II myosin genes are required for septation, development, and pathogenicity in *F. graminearum* (Song et al., 2013), *A. nidulans* (Taheri-Talesh et al., 2012), and *A. fumigatus* (Renshaw et al., 2016). Myosin V proteins are double-headed motors that mediate vesicle transportation and are important for maintaining cell polarity (Johnston et al., 1991; Govindan et al., 1995; Hammer and Sellers, 2011). Previous studies have suggested that class V myosin is essential for tip growth, cellular polarization, and virulence of pathogenic fungi, such as *C. albicans* (Woo et al., 2003), *A. nidulans* (Zhang et al., 2011; Taheri-Talesh et al., 2012), *Ustilago maydis* (Weber et al., 2003) and *A. fumigatus* (Renshaw et al., 2016; Renshaw et al., 2018). Chitin synthase enzymes with myosin motor domains (Chs-MMD) found specifically in filamentous fungi are critical in promoting the synthesis of chitin at the hyphal tip, thus, influencing fungal growth and the architecture of fungal infection structures. Almost all mutant disrupted in Chs-MMD genes in *Aspergillus, Fusarium, Penicillium digitatum, N. crassa*, and *U. maydis* feature morphological aberrations and reduced conidiation. These enzymes are also involved in the osmo-tolerance and tolerance to hydrogen peroxide, and the ability to grow at 37°C. These attributes are crucial for virulence and the host infection process (Fernandes et al., 2016). These collective findings strongly indicate myosin proteins are essential in a variety of cellular processes during fungal development and pathogenicity.

Regardless of available data from previous studies, the functions of myosin motors, and in particular the myosin II heavy chain in Mucoralean fungi causing mucormycosis remain understudied. In this study, to explore the possible roles of myosin II proteins in *M. circinelloides*, an initial functional screening was conducted using the RNAi approach. Gene deletions were then performed, followed by *in vivo* virulence assays of knockout mutant strains in the invertebrate model *Galleria mellonella* to investigate the impact of these motor proteins on the pathogenic potential of the fungus.

## Results

### Identification of myosin II homologs in *Mucor circinelloides*

To identify myosin II proteins and determine if there were additional unidentified myosin heavy chain genes in *M. circinelloides*, we carried out BLAST searches of the *M. circinelloides* genome databases at The Fungal Genomics Resource of the Joint Genome Institute (MyCocosm, JGI), with *A. nidulans* myosin sequences retrieved from protein database of National Center for Biotechnology (NCBI) (see **Table S1** for accession numbers). Seventeen myosin homologs were identified from the BLASTP searches, with their encoding genes distributed randomly in the genome of *M. circinelloides* (**Table S2**). Analyses using InterProScan on these 17 sequences revealed two proteins (IDs of 149958 and 136314) containing distinctive conserved domains of class II myosin heavy chain. Protein IDs 149958 and 136314 were predicted to have a length of 1136 and 1006 amino acids, respectively. They both contain an N-terminal myosin motor head domain, an IQ calmodulin-binding motif, and a C-terminal tail domain with a coiled-coil morphology. Interestingly, while protein ID 149958 has a myosin-II distinctive SH3-like domain, ID 136314 does not contain this domain in its structure (**Figure S1**). Phylogenetic analysis also showed that these two proteins fit perfectly among the clade of previously described fungal type II myosin motors (**Figure S2**), leaving no doubt that both proteins are type II myosin heavy chains. Hence, the ID 149958 and 136314 homologs were designated Myo2pA (encoding by gene *mcmyo2A*) and Myo2pB (encoding by gene *mcmyo2B*), respectively.

### Myosin II-silencing phenotypes display filamentous growth defect and sporulation reduction of *Mucor circinelloides*

**Figure 1:**
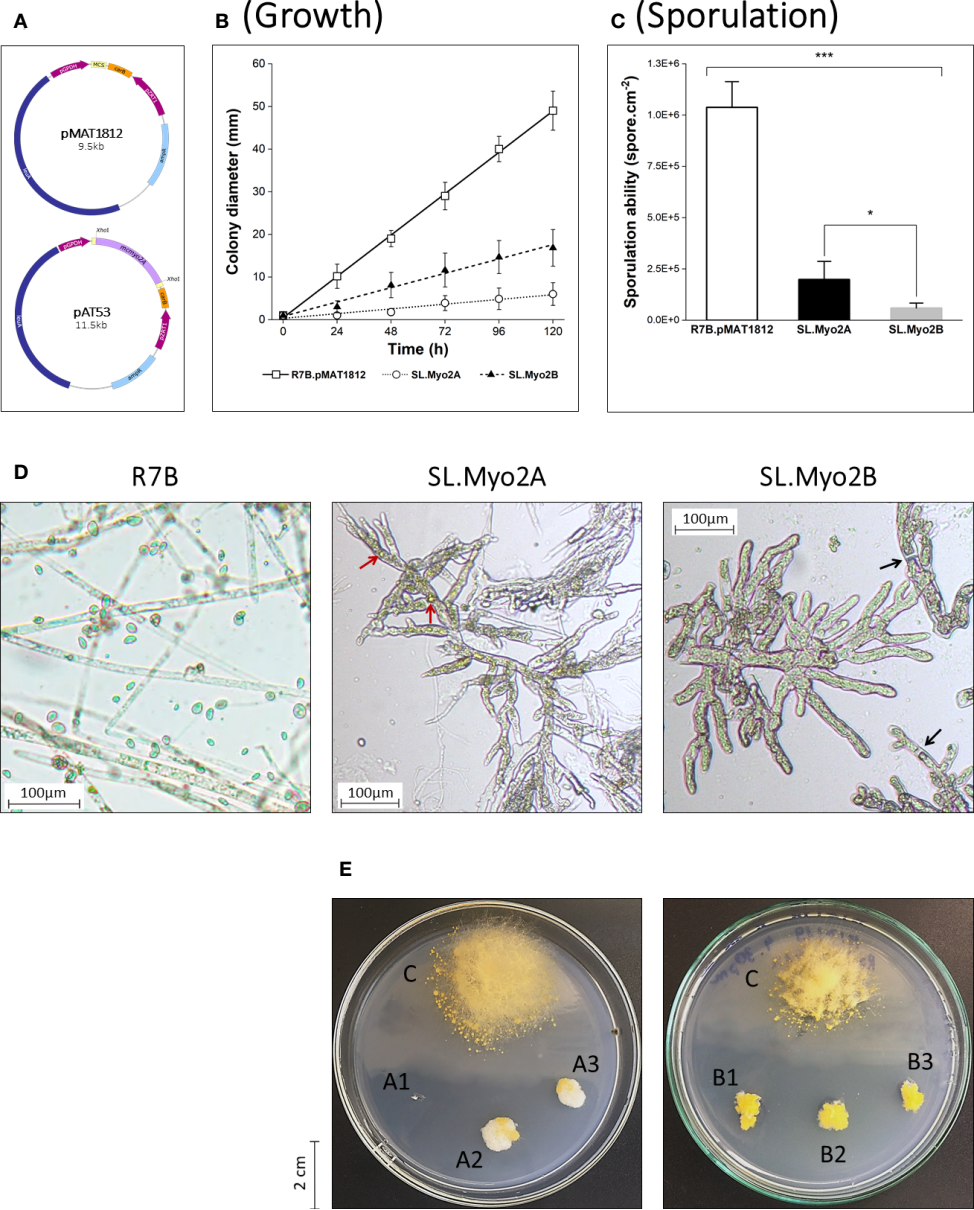
Initial functional screening of myosin II motors in *Mucor circinelloides via* RNA interference. **(A)** Schematic illustration of vector pMAT1812 and the RNAi plasmid pAT53 corresponding to the *mcmyo2A* gene. Quantification of growth rate **(B)** and vegetative sporulation **(C)** of myosin II silencing phenotypes (*p<0.05, ***p<0.001). Small pieces of grown mycelia of each strain were grown in YNB medium plates (pH 2.8) at 26°C under a continuous light condition for 120 hours in the growth rate measurements. In the case of sporulation assessment, the inoculated fungi were incubated for 72 hours to collect the total vegetative spores. **(D)** Microscopic images display morphological abnormalities of myosin II silencing phenotypes, compared to the wild-type (WT) R7B. Red arrows denote pigment-filled vesicles; black arrows denote septa in fungal hyphae. Some elongated WT-like hyphae can be observed in SL.Myo2A (background) due to the decrease of silencing effect through generations in the silencing mutant. **(E)** Colonies of SL.Myo2A (A1–3) and SL.Myo2B (B1–3), compared to the control R7B.pMAT1812 strain **(C)** at 72 h of incubation. Colonies were grown on YNB media (pH 2.8) at 26°C under a continuous light condition.

To perform an initial functional screening of myosin II proteins in *M. circinelloides*, we previously generated two RNAi plasmids (pAT53 and pAT52), corresponding to *mcmyo2A* and *mcmyo2B*, respectively (Le et al., 2020a; Le et al., 2020b). These dsRNA-expressing plasmids constructed from the pMAT1812 vector contain fragments of the target genes and are flanked by two opposite promoters that trigger the silencing mechanism of *M. circinelloides* (**Figure 1A**). These RNAi plasmids were transformed into protoplasts of the wild-type R7B strain (*leu*A^-^) using electroporation method. The control strain was the wild type containing the empty vector pMAT1812 (R7B.pMAT1812). The silencing mutants (SL.Myo2A and SL.Myo2B) displayed a significant reduction in filamentous growth. The relative growth rate of these two mutants was three times (SL.Myo2B) and even 8 to 10 times (SL.Myo2A) lower than the R7B.pMAT1812 strain (**Figures 1B, E**). Furthermore, the disruption of myosin II-encoding genes resulted in declined sporulation. Compared to the control strain, the sporulation abilities of SL.Myo2A and SL.Myo2B were only nearly 20% and 6%, respectively (**Figure 1C**). Regarding the morphology, the silencing mutants showed abnormally short, inflated, and highly branched hyphae (**Figure 1D**). In addition, the morphology of sporangia was aberrant. It is worth noting that some wild-type-like (WT-like) hyphae can be observed in the silencing mutants (**Figure 1D** – SL.Myo2A). The RNAi efficiency does not spread equally among the silencing phenotypes, especially in filamentous fungi which contain many nuclei inside a hypha. In addition, RNA interference has been considered a post-transcriptional gene silencing mechanism, suppressing gene expression by the degradation of homologous mRNA. This effect is not inherited but instead is decreased through generations (Ruiz-Vázquez et al., 2015; Torres-Martínez and Ruiz-Vázquez, 2016). Hence, during the development, the silencing effect had been reversed in a part of the hyphae, resulting in normal/WT-like phenotypes that could be observed. The collective results suggest that myosin II proteins could play a critical role in the filamentous growth, sporulation, and morphology of *M. circinelloides*.

### Generation of myosin II knockout mutant strains in *M. circinelloides*

**Figure 2:**
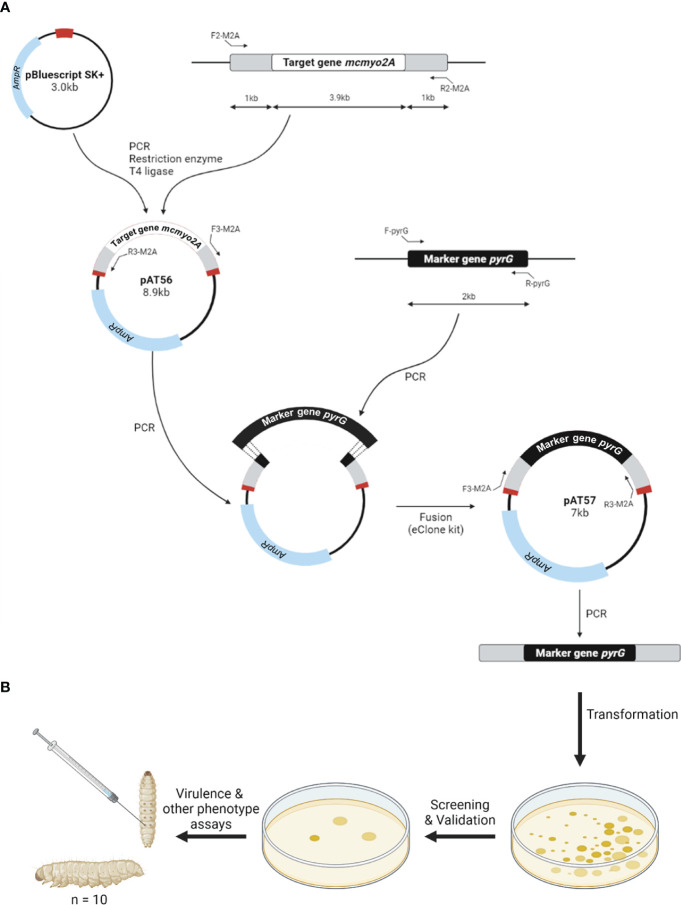
Schematic diagram of the construction of gene disruption cassettes **(A)** and phenotypic analyses of knockout mutants **(B)**.

Following the initial assessment using the gene silencing approach, gene knockout was performed to further investigate the impact of myosin II proteins in *M. circinelloides*. Myosin II encoding genes were first cloned to create disruption cassettes that could promote gene replacement *via* the double-crossover mechanism. Null mutants were generated using electroporation transformation and were validated with the selection process. The workflow of gene disruption and phenotypic analyses of knockout mutants was illustrated in **Figure 2**.

**Figure 3:**
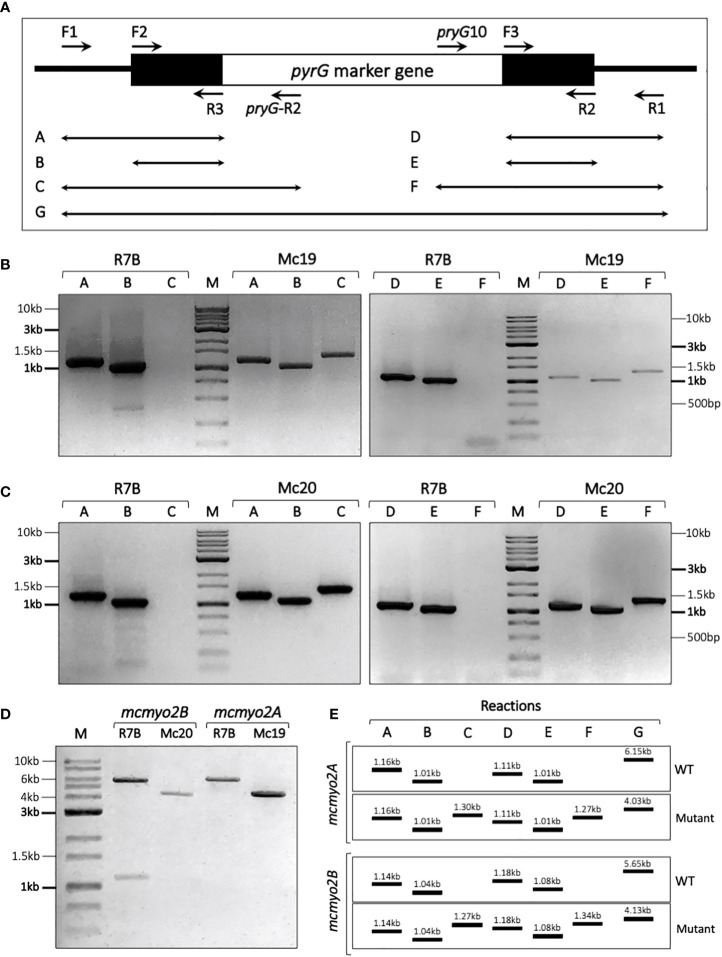
Verification of transformants using PCR-based strategy. **(A)** Schematic diagram illustrating the location of primers along the disruption cassette in the replacement site and the combination of primers used in each PCR reaction. The black line denotes the replacement site. The black boxes denote the upstream and downstream segments of the target gene, and the white box denotes the marker gene *pyrG* in the disruption cassette. **(E)** Expecting results of validating PCR reactions. Validation of transformants corresponding to *mcmyo2A* ***(*B*)*** and *mcmyo2B* **(C)**, compared to the control R7B strain (wild-type). **(D)** Results of PCR (reaction G) to check the genotypes of the transformant.

The PCR-based technique was used to verify the replacement sites, as well as the genotype (homokaryon or heterokaryon) of these mutants (**Figure 3**). The two transformants selected from *mcmyo2A* and *mcmyo2B* disruption only harbored the DNA fragments corresponding to the correct integration of the disruption fragment at the corresponding loci (**Figure 3D**), indicating that they were homokaryons for the mutant allele. Both myosin II encoding genes could be completely deleted from the genome of *M. circinelloides*. The validated knockout mutant strains were designated Mc19 (Δ*mcmyo2A*) and Mc20 (Δ*mcmyo2B*). The described PCR-based strategy we applied helped confirm the replacement of the target genes with the selection marker (*pyrG*) and check if the mutants were homokaryon or heterokaryon. However, this strategy could not detect ectopic insertion that could occur during the integration of the disruption cassette. Theoretically, the possibility of a simultaneous occurrence of correct replacement of the target gene and ectopic insertions is relatively low. A study on *N. crassa* also suggests that nuclei which undergo transformation by homologous recombination are not highly competent at ectopic integration (Miao et al., 1995). Despite a low frequency, it is worth noting that transformants with multiple copies of the disruption cassette could be produced during the generation of knockout mutants in this study.

### Disruption of myosin II encoding genes significantly reduces the polarity growth and sporulation and affects the morphology of *M. circinelloides*

**Figure 4:**
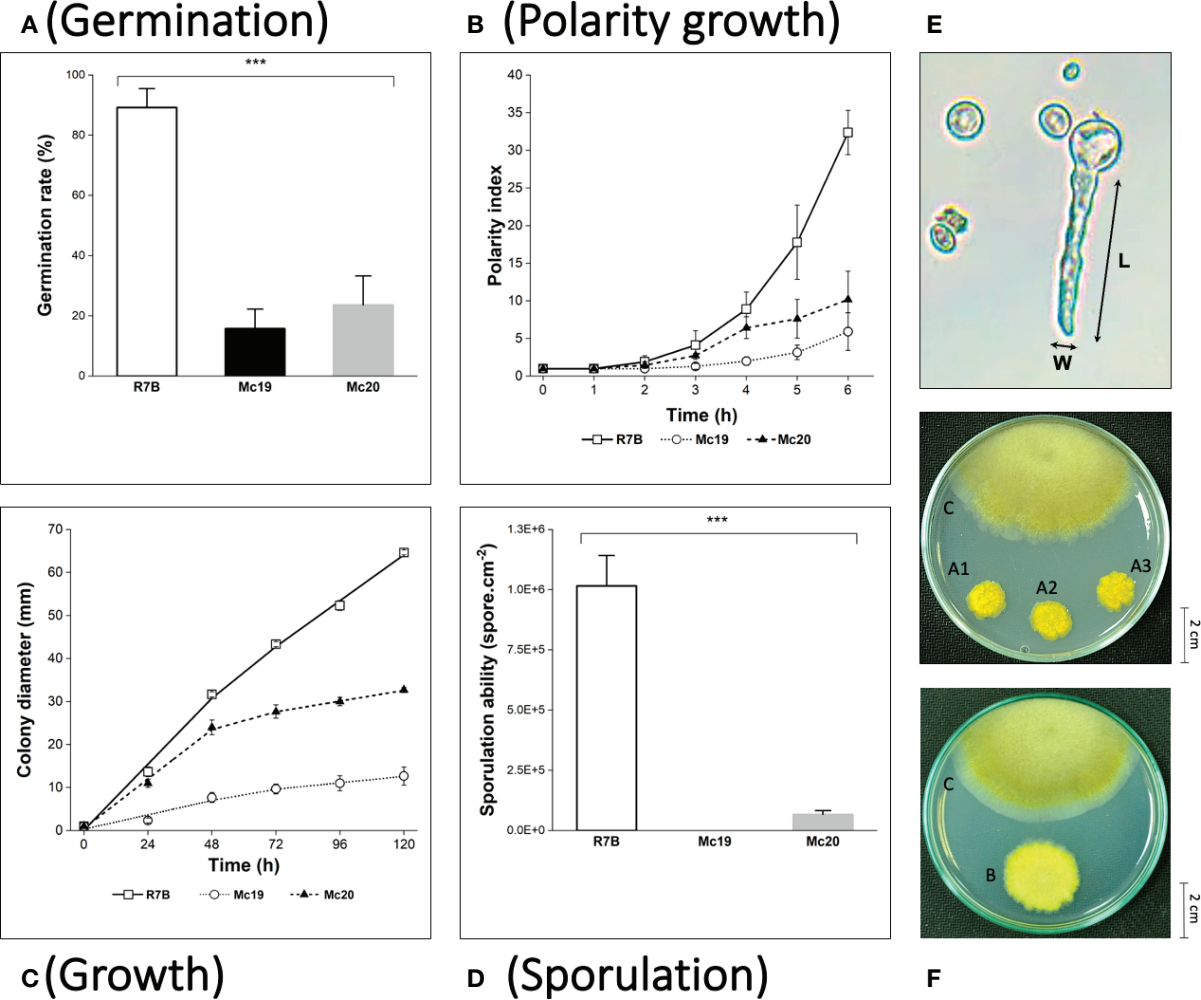
Phenotypic analyses of the myosin II null mutants Δ*mcmyo2A* (Mc19) and Δ*mcmyo2B* (Mc20). The analysis includes quantification of germination at the 6 h of incubation in MMC liquid media (pH 4.5) **(A)**, polarity growth **(B)**, hyphal growth **(C)**, and vegetative sporulation **(D)** (***p<0.001). **(E)** The calculation of the polarity index representing polarity growth: cell length (L) divided by cell width (W). Small pieces of grown mycelia of each strain were grown in MMC medium plates (pH 3.2) at 26°C under a continuous light condition for 120 hours in the growth rate measurements. In the case of sporulation assessment, the inoculated fungi were incubated for 72 hours to collect the total vegetative spores. **(F)** Colonies of Mc19 (A1–3) and Mc20 **(B)**, compared to the control R7B strain **(C)** at 96 h of incubation. Colonies were grown on MMC media (pH 3.2) at 26°C under a continuous light condition.

The polarity index (the ratio of the cell length divided by the cell width, **Figure 4E**; (Schuster et al., 2012) is an indicator of germination and hyphae formation ability. The index has been associated with fungal growth and virulence. In the early growth stage of *M. circinelloides* in liquid medium (from 0 to 12 hours of culture), in which the vegetative spores germinate and develop hyphae (Lubbehusen et al., 2003), the knockout mutants showed remarkably suppressed polarity indices compared to the control wild-type strain (**Figure 4B**). In the wild-type R7B strain, during the first hours, the vegetative spores increased in size due to water and nutrition absorbance. Beginning at 3 h, the spores of the control strain started to germinate and rapidly developed elongated hyphae for the next several hours. On the contrary, the spores of the Mc19 and Mc20 strains showed notably delayed germination and the majority exhibited either yeast-like or isotropic growth, leading to considerably low polarization (**Figure 4B**).

**Figure 5:**
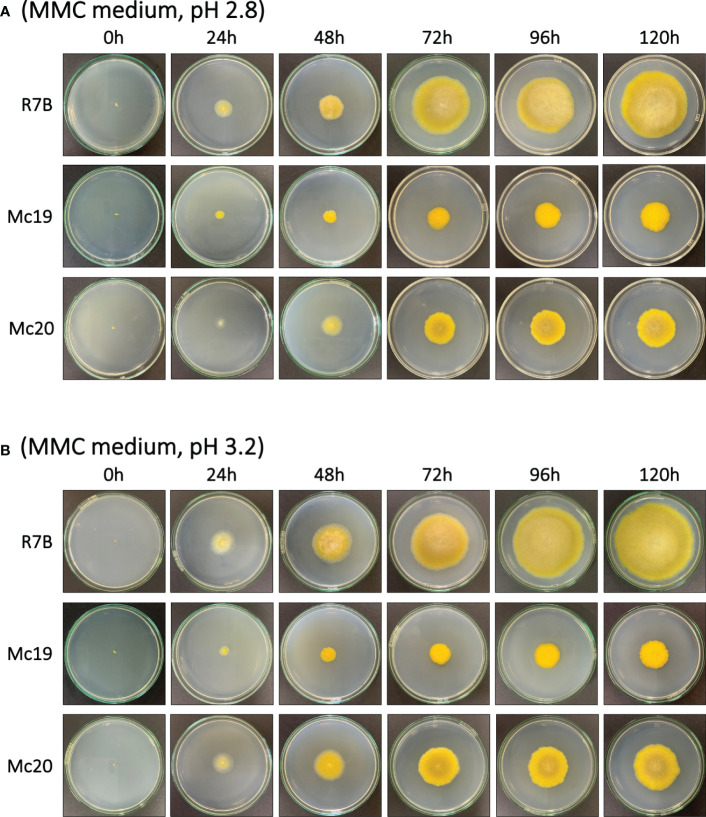
The vegetative growth of the myosin II null mutants Δmcmyo2A (Mc19) and Δmcmyo2B (Mc20) compared to the wild-type strain (R7B). Colonies were grown on MMC medium at 26ºC under a continuous light condition, from 0 h to 120 h, with pH 2.8 **(A)** and pH 3.2 **(B)**.

**Figure 6:**
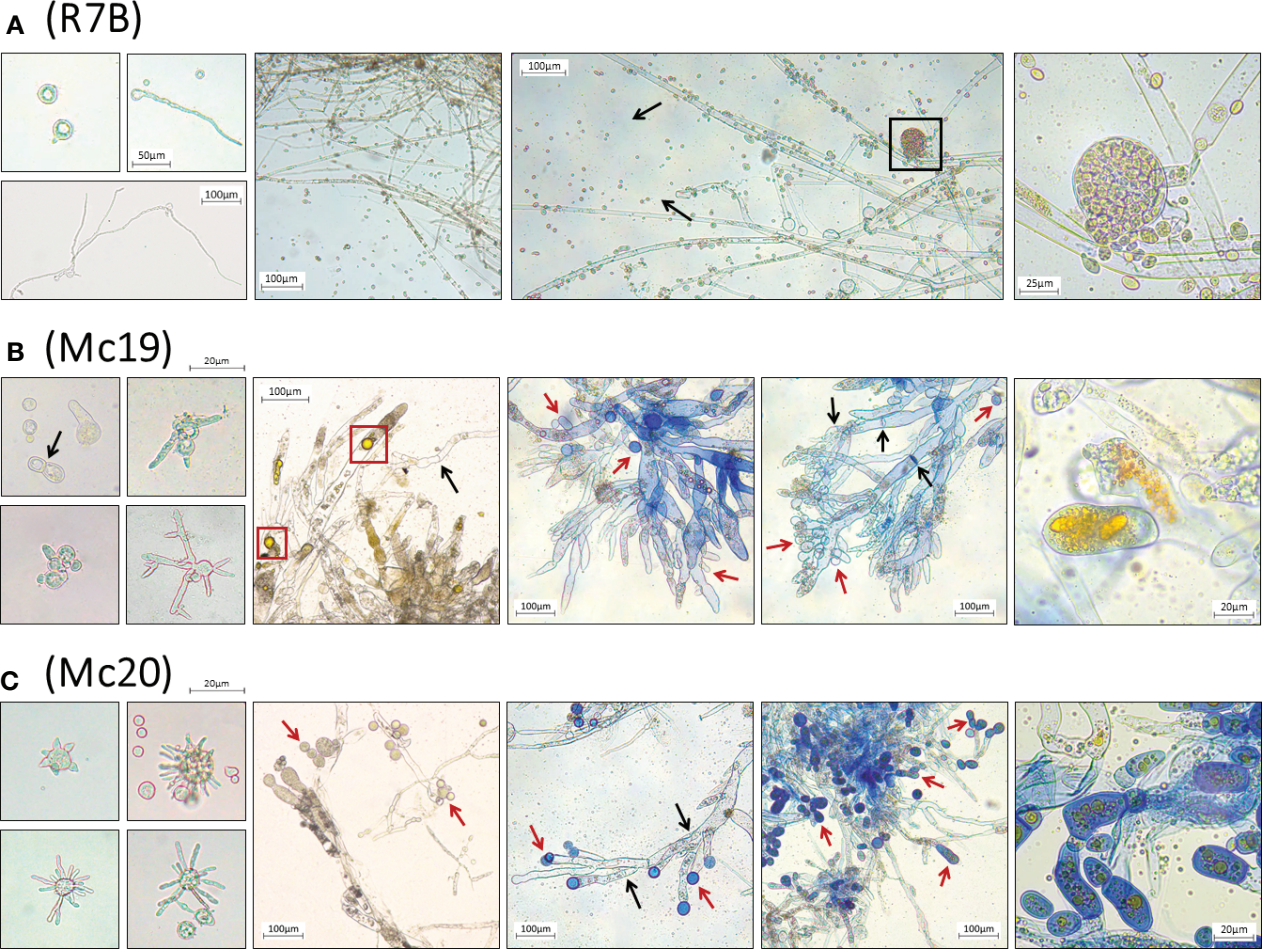
Microscopic images of morphological abnormalities of vegetative spores during germination, yeast-like cells, and fungal hyphae of the myosin II null mutant Δ*mcmyo2A* **(B)** and Δ*mcmyo2B* **(C)**, compared to the hyphae and the sporangia containing vegetative spores of the wild-type R7B **(A)**. The disruption of myosin II encoding genes led to a declination of sporulation. The mutants generate yeast-like cells, which locate in the hyphal tips and are separated from the hyphae by pseudo septa. The yeast-like cells and fungal hyphae of the mutants contain numerous pigment-filled vesicles. The black box denotes the sporangium containing vegetative spores; red boxes denote the pigment-filled vesicles in fungal cytoplasm. Black arrows point to the septa in fungal hyphae; red arrows point to the yeast-like cells of the mutants. The fresh vegetative spores were cultured in MMC liquid media (pH 3.2), while separated colonies of different strains grown on MMC solid media (pH 3.2) provided the mycelia to make specimens for microscopic observations.

The results of the evaluations of the growth of mutant strains in solid medium were highly consistent with the observations of the silencing phenotypes. These findings again indicated the crucial contributions of the myosin II encoding genes in *M. circinelloides*. As expected, knockout mutants displayed decreased growth rate (**Figure 4C** and **Figure 5**) and a strongly diminished sporulation (**Figure 4D**). At 120 h of culture, the colony diameter of the wild-type R7B control was twice that of the diameter of the Δ*mcmyo2B* strain and five times that of the Δ*mcmyo2A* mutant. Interestingly, both mutants developed rather faster in the first 48 hours, but this pace in the Mc20 strain (Δ*mcmyo2B*) slowed down upon further incubation (**Figure 4C** and **Figure 5**). In addition, after 48 h the mutant strains displayed abnormally short, septate, and branched filaments with inflated hyphae tips containing large pigment-filled vesicles (**Figure 6**), resulting in an unusual yellowish color of the fungal colonies (**Figure 4F** and **Figure 5**). Deletion of *mcmyo2A* and *mcmyo2B* was likely to disable the sporulation of the fungus as the relative sporulation ability of the Mc20 was just approximately 7%, while Mc19 almost completely lost the capacity to sporulate (**Figure 4D** **)**. Notably, the disruption of myosin II encoding genes prevented the formation of sporangia that produce vegetative spores, leading to the diminished sporulation. Instead, the mutants generated yeast-like cells, which were located in the hyphal tips and are separated from the hyphae by pseudo septa (**Figures 6B, C**). Taken together, these results suggest that myosin-II proteins play important roles in the development and morphology of *M. circinelloides.*

### Myosin II proteins are essential for pathogenicity of *Mucor circinelloides*

*In vivo* virulence assessments of myosin-II mutant strains were performed in the invertebrate larvae *Galleria mellonella* – a common infection model for mucormycosis study (Jacobsen, 2019), using inoculation of either 5,000 or 10,000 vegetative spores or yeast-like cells per dose (the Δ*mcmyo2A* mutant was unable to produce enough spores, so yeast-like cells were used instead for these tests). Before the actual assays, the concentration of fungal inoculum and testing conditions were adjusted and optimized with sporangiospores of virulent wild-type strain R7B, specifically for this study, to achieve a substantial killing rate at 75% and 90% lethal doses (LD75, equivalent to 5,000 spores per dose and LD90, equivalent to 10,000 spores per dose) (Durieux et al., 2021). The survival of infected larvae was monitored every 24 hours for 6 days consecutively. A group of non-injection individuals and the other group including larvae injected with phosphate-buffered saline PBS were monitored as controls. The Mc19 strain (Δ*mcmyo2A*) appeared avirulent as the survivor rate of infected larvae showed no significant difference with control groups at both injection concentrations (p=0.9998 and p=0.9871, respectively) (**Figure 6**). On the other hand, the vitality of the hosts infected with the Mc20 mutant (Δ*mcmyo2B*) remained the same to that of group injected with spores of wild-type strain at either LD75 or LD90 dose (p=0.2854 and p=0.9051, respectively), suggested that the *mcmyo2B* gene was less likely to be relevant to the pathogenesis of *M. circinelloides* despite the delayed growth, sporulation reduction, and abnormal morphology of hyphae.

## Discussion

Myosin is a remarkable superfamily of molecular motors that use the energy derived from ATP hydrolysis to produce forces to power movement on actin filaments in all eukaryotic cells (Sweeney et al., 2020). Its members are currently grouped into many classes (Odronitz and Kollmar, 2007; Hartman and Spudich, 2012; Sebé-Pedrós et al., 2014), with diverse types of cellular functions. In fungi, four classes of myosin have been found: myosin I, myosin II, myosin V, and fungus-specific chitin synthase (Chs) with myosin motor domains (Xiang and Plamann, 2003). Previous studies showed that generally there are four to six myosin motors, with only one or two members in each of these four classes, found in classical fungal models. *Saccharomyces cerevisiae* contains five myosin proteins belonging to three classes (Watts et al., 1987; Johnston et al., 1991; Haarer et al., 1994; Goodson and Spudich, 1995; Geli and Riezman, 1996), while *A. nidulans* possesses three myosin encoding genes encompassing three classes (McGoldrick et al., 1995; Taheri-Talesh et al., 2012) and two Chs-MMDs (Fujiwara et al., 1997; Takeshita et al., 2006). Similarly, myosin motors classified among three types of fungal myosin were discovered and characterized in *C. albicans* (Oberholzer et al., 2002; Woo et al., 2003)*, N. crassa* (Calvert et al., 2011; Fernando et al., 2016; Ramírez-del Villar et al., 2019), and *U. maydis* (Weber et al., 2003; Banuett et al., 2008). However, as indicated, in the genome of *M. circinelloides*, 17 putative myosin homologs were identified that displayed a significantly higher abundance of myosin motor molecules in comparison to other fungi (**Table S1** and **Table S2**). They include two homologs belonging to class I myosin, two type II myosin heavy chains, four proteins classified as myosin V, and six proteins that are homologs of the Chs-MMD. In addition, there are three sequences, which are much shorter and lack the structure of a classic myosin protein. We considered them as short isoforms, but not members of myosin motors in *M. circinelloides* (**Table S2** and **Figure S2**). Interestingly, in a study on the evolution of myosin V-based organelle inheritance, seven putative myosin class V proteins were found in *Rhizopus oryzae*, another member of the order Mucorales (Mast et al., 2012). The abundance of myosin motors in Mucoralean fungi might be explained by the differences in taxonomy between these species and other fungal models. The fungus *M. circinelloides* and other Mucoralean fungi are classified among the zygomycetous fungi Mucoromycota, a phylum in the basal fungal lineages (Spatafora et al., 2016; Wijayawardene et al., 2018). In contrast, *S. cerevisiae, Aspergillus, Candida*, and *Neurospora* species are members of Ascomycota and the plant pathogenic *U. maydis* belongs to Basidiomycota, two phyla of higher fungi Dikarya (Hibbett et al., 2018). Noticeably, the present findings are consistent with the results from the study of Liu et al. (2017) in the evolution of the fungal chitin synthase gene family, as fungi in early-diverging Mucoromycota possess multiple chitin synthases with myosin head, whereas most members of Ascomycota and Basidiomycota contain none or only one to two homologs of the Chs-MMD (R. Liu et al., 2017). These results indicate that a large number of myosin motor molecules are required for the growth, development, and other important biological processes in basal fungi, including Mucoralean fungi.

**Figure 7:**
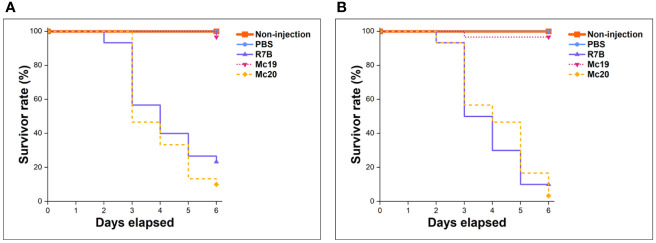
Results of virulence assays of myosin II mutants in the *Galleria mellonella* invertebrate model. Injections contain 5,000 vegetative spores or yeast cells per dose **(A)**, and 10,000 spores/yeast cells per dose **(B)**.

Fungal myosin motors are involved in actin organization, endocytosis, cytokinesis, vesicle/organelle transportation, and polarized cell wall synthesis (Xiang and Plamann, 2003; Xiang and Oakley, 2010). In this study, we performed the first identification and functional characterization of type II myosin genes from a member of Mucorales fungi. The obtained results show that the *Mucor* myosin class II motors regulate growth, morphology, and contribute to the pathogenicity of the fungus by being involved in cytokinesis and polarity during hyphal growth. Myosin II null mutant strains displayed a significantly reduced growth rate, impaired sporulation, and abnormal morphological hyphae compared with the wild-type strain. However, some remarkable differences were apparent between the influences of the two myosin II encoding genes in *M. circinelloides*. Myo2pA is likely to be more important as both silencing phenotype and null mutant are highly affected in terms of germination, polarization, and growth rate. Additionally, the disruption of *mcmyo2A* was likely to completely disable sporulation, while Δ*mcmyo2B* remained able to produce spores. *In vivo* virulence assays of myosin II mutant strains performed in the invertebrate model *G. mellonella* indicated that the *mcmyo2A*-knockout strain was avirulent, whereas the pathogenesis of the *mcmyo2B* null mutant was likely to remain unaltered (**Figure 7**). The lack of the Src homology 3 (SH3) domain in the putative structure of Myo2pB (**Figure S1**) might be responsible for these functional differences. Mediating protein-protein interactions through binding to proline-rich stretches, the SH3 domain is present in various proteins that associate with the actin cytoskeletal organization and with signal transduction (Kuriyan and Cowburn, 1997). In class II myosin motors, SH3 domains are located at their N-terminal head, proximal to the essential light chain (ELC). Together with the regulatory light chain (RLC), the domains involve in stabilization of the C-terminal α-helical region in the heavy chain (Rayment et al., 1993). In addition, the SH3 domain interacts with the N-terminal proline-rich extension of the ELC during the ATPase cycle, modulating the kinetics of myosin II (Lowey et al., 2007). The absence of this critical domain possibly causes defects in the stability and mobility of Myo2pB, which lessens its influence compared to the Myo2pA heavy chain. It is also possible that *mcmyo2A* and *mcmyo2B* were expressed at different levels in our experimental condition. An in-depth gene expression analysis is required to more accurately evaluate the functional features of type II myosin encoding genes.

Our findings are generally consistent with the limited data available from other fungi. Knockdown expression and deletion of both *mcmyo2A* and *mcmyo2B* resulted in delayed germination as well as significant reductions in hyphal growth and sporulation (**Figures 4**, **5**). In addition, type II myosin heavy chains play critical roles in the morphogenesis of *M. circinelloides*, as the lack of either *mcmyo2A* or *mcmyo2B* leads to distorted hyphal growth. The display of isotropic growth during the early growth stage as well as the morphology of short and inflated hyphae during the later growth stage of the null mutants indicate that class II myosin motors are important to the cell polarization. The reduction of polarized growth could be explained by the lack of transporters that deliver the secretory vesicles to the growth regions (Steinberg, 2011). It is possible that some of the vesicles, which are specific cargoes of Myo2pA and Myo2pB in *Mucor*, constitute the Spitzenkörper (apical body), a structure found in the tip of fungal hyphae that acts as the organizing center for hyphal growth and morphogenesis. In filamentous fungi, the apical body consists of many small vesicles and is present in growing hyphal tips during spore germination, and is where branch formation occurs (Bartnicki-García, 2002). Its position in the hyphal tip correlates with the direction of hyphal growth. Thus, the lack of the appropriate vesicle concentration at specific points during germination of Δ*mcmyo2A* and Δ*mcmyo2B* mutants should avoid their polarized hyphal growth. Another hypothesis is that the *Mucor* myosin II heavy chains regulate tip growth through interaction with other motors and molecules, which are primarily responsible for cell polarization. Interestingly, while type II myosin is necessary for the side branching in *A. nidulans* (Taheri-Talesh et al., 2012), disruption of myosin II encoding genes reduces the hyphal elongation and strongly induces the branching in *M. circinelloides* (**Figures 6B, C**). Strikingly, although the *Mucor* fungus is aseptate, null mutants of either *mcmyo2A* or *mcmyo2B* showed random septum formation in their hyphae (**Figures 6B, C**). Instead of forming sporangia containing vegetative spores as the wild-type strain (**Figure 6A**), the mutants generated yeast-like cells, which located in the hyphal tips and are separated from the hyphae by pseudo septa (**Figures 6B, C**). It is partly reminiscent of the roles of myosin II proteins in the fission yeast *Schizosaccharomyces pombe* and budding yeast *S. cerevisiae*. The absence of either *myo2^+^* or *myp2^+^*, two myosin II genes in *S.pombe*, results in elongated, multiseptated, and occasionally branched cells. In contrast, overexpression of these genes is harmful to cell growth, producing multinucleated cells that lack septa and no actin ring formation (Kitayama et al., 1997; Bezanilla et al., 1997). Similarly, in *S. cerevisiae*, the mutation of type II myosin gene *MYO1* leads to changes in the budding pattern as the mutant either grows as an interconnected chain of cells or shows aberrant septum formation (Tolliday et al., 2003). On the contrary, the lack of type II myosin disrupts the septation in filamentous fungi such as *F. graminearum* (Song et al., 2013)*, A. nidulans* (Taheri-Talesh et al., 2012), and *A. fumigatus* (Renshaw et al., 2016). Regarding structural features, the discovery of conserved domains and motifs, followed by phylogenetic analysis on described fungal type II myosin proteins, revealed that Myo2pA and Myo2pB sequences in *M. circinelloides* are considerably shorter in length. Furthermore, rather than homologs of yeast or filamentous fungi, they closely resemble the type II myosin from the dimorphic fungus *U. maydis* (**Figure S1**) despite the distance in taxonomy. According to protein motifs discovery results in yeasts and dimorphic fungi, such as the *Mucor* species, type II myosin motors lack several distinctive motifs at the C-terminal end compared to myosin homologs in filamentous fungi. These C-terminal motifs could constitute or serve as organelle/vesicle-specific receptors that are parts of the cargo-binding domain of the myosin motor, which have a major role in the regulation of motor–cargo interaction. Supporting this theory, studies on type V myosin heavy chain Myo2p in *S. cerevisiae* revealed that different parts of the protein are responsible for binding to different vesicles (Pashkova et al., 2006; Tang et al., 2019). The differences in the composition of protein motifs suggest that myosin type II heavy chains, and myosin motors in general, are crucial and specific for the type of growth in fungi, as myosin II members play a major role in cytokinesis in yeasts (Xiang and Oakley, 2010), whereas in filamentous fungi, type II myosin proteins are essential for septation (Taheri-Talesh et al., 2012; Song et al., 2013; Renshaw et al., 2016). Like the dimorphic fungi *U. maydis*, the switch from yeast to a tip-growing hypha is important to the pathogenicity of the *Mucor* fungus. A yeast-like phenotype is avirulent, while hyphal growth induces fungal virulence (Lee et al., 2013). Indeed, we observed that the deletion of *mcmyo2A* led to the loss of pathogenicity in the *Mucor* species in the *G. mellonella* invertebrate model (**Figure 7**). It is also noteworthy that myosin II proteins could involve in pigment transportation in *M. circinelloides* as the accumulation of large vesicles containing yellow pigments, probably carotenoids, was observed in the cytoplasm of mutant cells (**Figures 6B, C**). Notably, our previous study on Myo5, one of the myosin class V proteins in *M. circinelloides*, which is encoded by the *mcmyo5* gene, showed very similar results (Trieu et al., 2017). Although the *mcmyo5* gene is indispensable, the absence of Myo5 protein inhibits hypha formation, leading to a yeast-like phenotype with diminished sporulation and excessive accumulation of carotenoids. Similar to the myosin II mutants, the hyphae of the heterokaryotic strain Δ*mcmyo5* ^(-)(+)^ appeared highly branched with septate tips. Assessments of *Mucor* virulence in the *G. mellonella* host system have shown a significant reduction in virulence of Δ*mcmyo5* ^(-)(+)^ mutant (Trieu et al., 2017). Obviously, type II and type V myosin motors in *M. circinelloides* are likely to be multitasking carriers and share various functions, involving polarity growth, branching pattern, abnormal septation, sporulation, pigment transportation, and pathogenicity. These facts also suggest a tight correlation between these two classes of myosin heavy chains in intracellular transportations related to the growth and morphogenesis of the *Mucor* fungus. Still, more observations need to be made to comprehensively understand working mechanisms, and in particularly, the coordination between myosin motors in *M. circinelloides.*

## Materials and methods

### Strains, growth, and transformation conditions

The leucine auxotroph R7B (*leuA* ^-^), derived from the *M. circinelloides f. lusitanicus* CBS277.49, was used as the wild-type strain. MU402 (*leuA^-^, pyrG^-^*) was the recipient strain for the generation of the knockout mutant (Vellanki et al., 2018).

The fungal cultures were grown at 26°C in minimum YNB (Yeast Nitrogenous Base) medium, the complete YPG (Yeast-Peptone-Glucose) medium, or selective MMC (Minimal Media with Casamino acids) medium with the pH adjusted to 4.5 and 3.2 for mycelial and colonial growth, respectively. Uridine (200 µg/mL) was added to the medium when required. In DNA recombinant transformation into protoplast, the particular medium was supplemented with 0.5M sorbitol to prevent cytolysis due to the osmotic gradient (Vellanki et al., 2018).

*Escherichia coli* strain DH5α grown at 37°C in the LB medium (pH 7–7.4) was used for all cloning experiments by applying the heat shock method (Froger and Hall, 2007). Medium for transformant selection was supplemented with ampicillin (100 µg/mL).

### Plasmids

To construct RNAi vectors with the target candidate genes, plasmid pMAT1812 was used as a cloning vector. Insert fragments corresponding to candidate genes were amplified with primers containing *Xho*I restriction sites to facilitate cloning into pMAT1812 (**Figure 1A**). Plasmid pAT53 contains a 2 kb fragment of gene *mcmyo2A* (ID 149958) was PCR-amplified using primer pairs M2F-SL1 and M2R-SL1 (**Table S3**). Plasmid pAT52 harbors a 0.9 kb fragment of gene *mcmyo2B* (ID 136314) was amplified by PCR reactions using primer pairs M2F-SL2 and M2R-SL2 (**Table S3**).

To create disruption cassettes for the target gene deletions, two cloning plasmids, pAT56 and pAT59, were first constructed using pBluescript SK+ as the template. These plasmids contain 5.9 kb and 5.5kb fragments corresponding to *mcmyo2A* and *mcmyo2B*, respectively, which cover the entire length of target genes and 1 kb sequences in each of their upstream and downstream regions amplified using pairs of primers F2-M2L1/R2-M2L1 and F2-M2L2/R2-M2L2 (**Figure 2A**). DNA insert fragments were cloned into pBluescript SK^+^ vector and introduced into *E. coli* DH5α cells. After the growth of transformants in the selective medium, colony PCR was used to rapidly detect individual colonies that contained the desired recombinant plasmids, using pair of primers F2-M2L1/R3-M2L1 and F2-M2L2/R3-M2L2 (**Table S3**). Plasmids purified from the positive clones were designated pAT56 and pAT59, and were verified using restriction enzyme digestions (**Figure S3A**). The sequences of the insert fragments were finally confirmed with DNA sequencing.

Next, linear vectors, approximately 5 kb in size, in which the sequences of target genes were removed but that still retained their upstream and downstream regions, were amplified using primers F3-M2L1/R3-M2L1 and F3-M2L2/R3-M2L2 with pAT56 and pAT59 as DNA templates, respectively. The primers (**Table S3**) contained short sequences (15 – 20bp) that are similar to the sequences at two ends of the *pyrG* gene. They enabled the *pyrG* fragment to be ligated with the linear vectors. Obtained linear vectors were ligated with a 2 kb fragment of selective marker gene *pyrG*, which was amplified using the primers F-pyrG and R-pyrG. The ligation mixtures were transformed into *E. coli* DH5α competent cells, followed by the selection and verification process using selective media, colony PCR, digestion with restriction enzymes (**Figure S3B**), and DNA sequencing. This results in two knockout vectors designated pAT57 and pAT60 (corresponding to *mcmyo2A* and *mcmyo2B*¸ respectively). Finally, specific linear disruption cassettes (approximately 4kb in size) were generated from pAT57 and pAT60 by PCR using F2-M2L1/R2-M2L1 or F2-M2L2/R2-M2L2 as primers (**Table S3**).

A complete description of the plasmids used in this work for cloning and functional analysis of the target genes can be found in **Table S4**.

### DNA manipulation and analysis

The sequences and characteristics of the *mcmyo2A* (ID 149958) and *mcmyo2B* (ID 136314) were retrieved from the genomic database of *M. circinelloides f. lusitanicus* CBS277.49 belonging to the MycoCosm (The Fungal Genomics Resource), Joint Genome Institute, US Department of Energy (DOE JGI) (Grigoriev et al., 2014).

The genomic DNA of *M. circinelloides* was extracted from mycelia using Fungi/Yeast Genomic DNA Isolation Kit (NORGEN Biotek, Ontario, Canada). Bacterial plasmid extractions were proceeded using the GenElute™ Plasmid Miniprep Kit (Sigma-Aldrich, Merck KGaA, Darmstadt, Germany), according to the manual of the manufacturer.

PCR reactions to amplified DNA fragments longer than 5 kb or requiring high precision were performed using Phusion™ HighFidelity DNA Polymerase (2 U/µL) (Thermo Fisher Scientific, Massachusetts, US). Shorter fragments were amplified using 2× PCR Master Mix (Thermo Fisher Scientific). PCR mixture preparations and thermocycling program settings were prepared according to the manufacturer’s guidelines.

PCR products and DNA samples were purified directly or by agarose 1% electrophoresis using GenElute™ PCR Clean-Up Kit or GenElute™ Gel Extraction Kit (Sigma-Aldrich). SimpliNano™ Spectrophotometer (Biochrom, Harvard Bioscience, UK) was used for DNA quantifications.

Digestion of DNA was facilitated using endonuclease restriction enzyme products (FastDigest) from Thermo Fisher Scientific. The DNA ligation utilized T4 DNA ligase 1 U/µL (Thermo Fisher Scientific) and the eClone kit (PHUSA BioChem, Vietnam) following the instructions of the manufacturers. Standard DNA recombinant manipulations were performed as described previously (Green and Sambrook, 2012).

### Mutant generation and verification

Electroporation transformation to generate mutants was performed as previously described (Gutiérrez et al., 2011).

For gene silencing, the wild-type R7B strain was used as the recipient strain. Transformants were selected in the YNB medium. the silencing phenotypes were selected based on the morphological differences between the transformants and the controls (R7B.pMAT1812) and/or the wild-type strain (R7B). In the RNAi plasmids, together with the insert fragments corresponding to candidate genes, there was carotenoid-producing gene *carB* positioned between two opposite promoters as a reporter gene (**Figure 1A**). The expression of these RNAi plasmids suppresses the production of carotenoids, and therefore, silencing phenotypes display white hyphae compared to the yellowish ones of the wild-type due to the lack of carotenoids. In addition, other abnormalities in the growth and morphology, such as the relative growth rate, the dimorphism, or the type of growth, were also considered to select targeted silencing phenotypes among transformants.

For gene knockout, the disruption cassettes were transformed into protoplasts of the MU402 strain (*pyrG^-^, leuA^-^*), triggering the double-crossover mechanism of gene replacement. The selection process proceeded on MMC selective medium (pH 2.8) for five to seven vegetative cycles to screen for abnormal phenotypes that might be the potentially desired mutants. Additionally, this process also aims to increase the proportion of transformed nuclei, as primary transformants are heterokaryons due to the presence of several nuclei in the protoplasts (Cervantes et al., 2013). A PCR-based strategy was applied to verify the genotype of transformants using different combinations of primers (**Figure 3 A** and **Table S3**). The results from PCR reactions would indicate whether the marker gene *pyrG* is correctly integrated into the target loci to successfully eliminate candidate genes and whether the selected transformants were homokaryon or heterokaryon.

### Phenotypic analysis and microscopic imaging

To measure the vegetative growth of strains and mutants, small pieces (approximately 1 mm in diameter) of grown mycelia were inoculated onto the surface of medium plates (8 cm in diameter). Vegetative growth was estimated by measuring the diameter of the colony using a ruler (Quiles-Rosillo et al., 2003) every 24 h for 5 consecutive days.

The measurement of sporulation ability was performed following the previously described procedure, using the calculation of spores harvesting from grown mycelia (Nicolás et al., 2008). Small pieces of mycelia (1-mm diameter) of the wild-type and mutant strains and mutants were transplanted to solid medium plates and were grown for 72 h at 26°C under continuous light condition. After the incubation, the diameter of each colony was determined. The complete colony was harvested in 1.0 mL sterile distilled water and rigorously vortexed to release the spores thoroughly. The spores were then counted using a hemocytometer. The sporulation ability is the ratio of the total number of spores divided by the surface area of the colony.

To assess the germination and the polarity growth, the fresh vegetative spores of each strain were cultured in 25 mL of MMC liquid medium (pH 4.5) in a sterile Erlenmeyer flask with the sporangial concentration adjusted to 10^6^ spores/mL. The Mc19 strain did not provide enough spore for the assay, therefore yeast-like cells with the same concentration (10^6^ yeast-like cells/mL) were used instead. Prepared Erlenmeyer flasks were kept overnight at 4°C, then changed to the growth condition at 26°C with constant shaking (200rpm) during seven hours. Every 1 h, temporary specimens were made, observed, and photographed using a computer-connected microscope system (ZEISS Axio Scope.A1 with Axiocam 105 Color) and ZEN Microscopy software 2.6 (Carl Zeiss, Oberkochen, Germany). With each sample (specimen), five snapshots were taken to capture the microscopic field of view at four corners and the center of the specimen. All snapshots were used to extract the data, including the total number of evaluated spores, the number of germinated spores, the length and width of fungal hyphae. Spores that produce germ tubes longer than their diameter were considered to have germinated. For the calculation of the polarity index, from each snapshot, ten germinating spores were measured using ImageJ software (a total of fifty germinating spores were measured each sample).

The virulence assays in *G. mellonella* were performed by injection of 10 μL of PBS containing vegetative spores or yeast-like cells into larvae (ten larvae per strain). The survival of infected larvae was monitored every 24 h for 6 consecutive days.

Each assay was conducted in three replications. The data were stored, analyzed, and illustrated using Microsoft Excel and Origin 2021b software. ANOVA analysis (α=0.05) followed by the Tukey *post hoc* test was applied to identify the statistically significant differences.

Regarding the determination of morphology, the spores and mycelia of mutant strains were observed and photographed using mentioned computer-connected microscope system (ZEISS Axio Scope.A1 with Axiocam 105 Color) and ZEN Microscopy software 2.6. The fresh vegetative spores were cultured in MMC liquid media (pH 3.2) with a sporangial concentration of 10^6^ spores or yeast-like cells per mL. Separated colonies of different strains grown on MMC solid medium (pH 3.2) provided the mycelia to make specimens for microscopic observations.

## Data availability statement

The original contributions presented in the study are included in the article/**Supplementary Material**. Further inquiries can be directed to the corresponding author.

## Author contributions

TT conceived the work and designed the experiments. ML and TT performed experiments and collected data. PN and ML analyzed the data. PN, TT and ML wrote the manuscript. TT, PN and HC revised the manuscript. All authors contributed to the article and approved the submitted version.
