# Supplementary material for: Myosin-II proteins are involved in the growth, morphogenesis, and virulence of the human pathogenic fungus Mucor circinelloides
Source: Front Cell Infect Microbiol. 2022 Dec 16;12:1031463. doi: 10.3389/fcimb.2022.1031463 (PMC9800795; doi:10.3389/fcimb.2022.1031463)
Supplement: Supplementary file 1 [file DataSheet_1.docx]

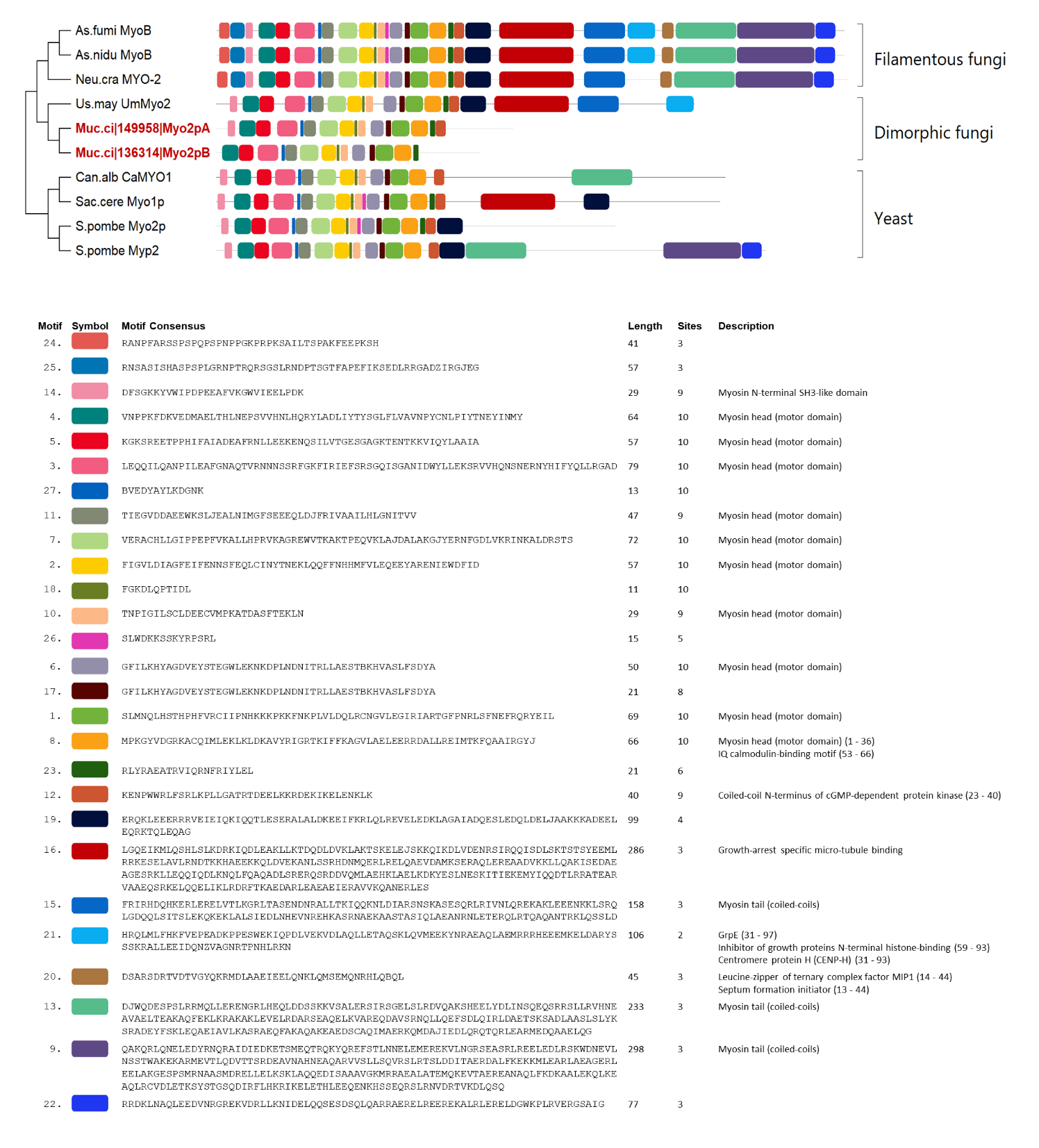
Supplementary Material

**Supplementary Figure 1.** Phylogenetic and motif discovery analysis of myosin-II homologs in *Mucor circinelloides* (Muc.ci) and pre-described myosin type II heavy chains in *Aspergillus fumigatus* (As.fumi), *A. nidulans* (As.nidu), *Neurospora crassa* (Neu.cra), *Ustilago maydis* (Us.may), *Candida albicans* (Can.alb), *Saccharomyces cerevisiae* (Sac.cere), and *Schizosaccharomyces pombe* (S. pombe). MEGA X was used to align the amino acid sequences of myosin-II homologs (with the ClustalW algorithm), and to construct a maximum-likelihood (ML) tree. Motif discovery was done using MEME Suit 5.4.1 and selected information of 27 discovered protein motifs is provided in the order of appearance in amino acid sequences (from left to right).


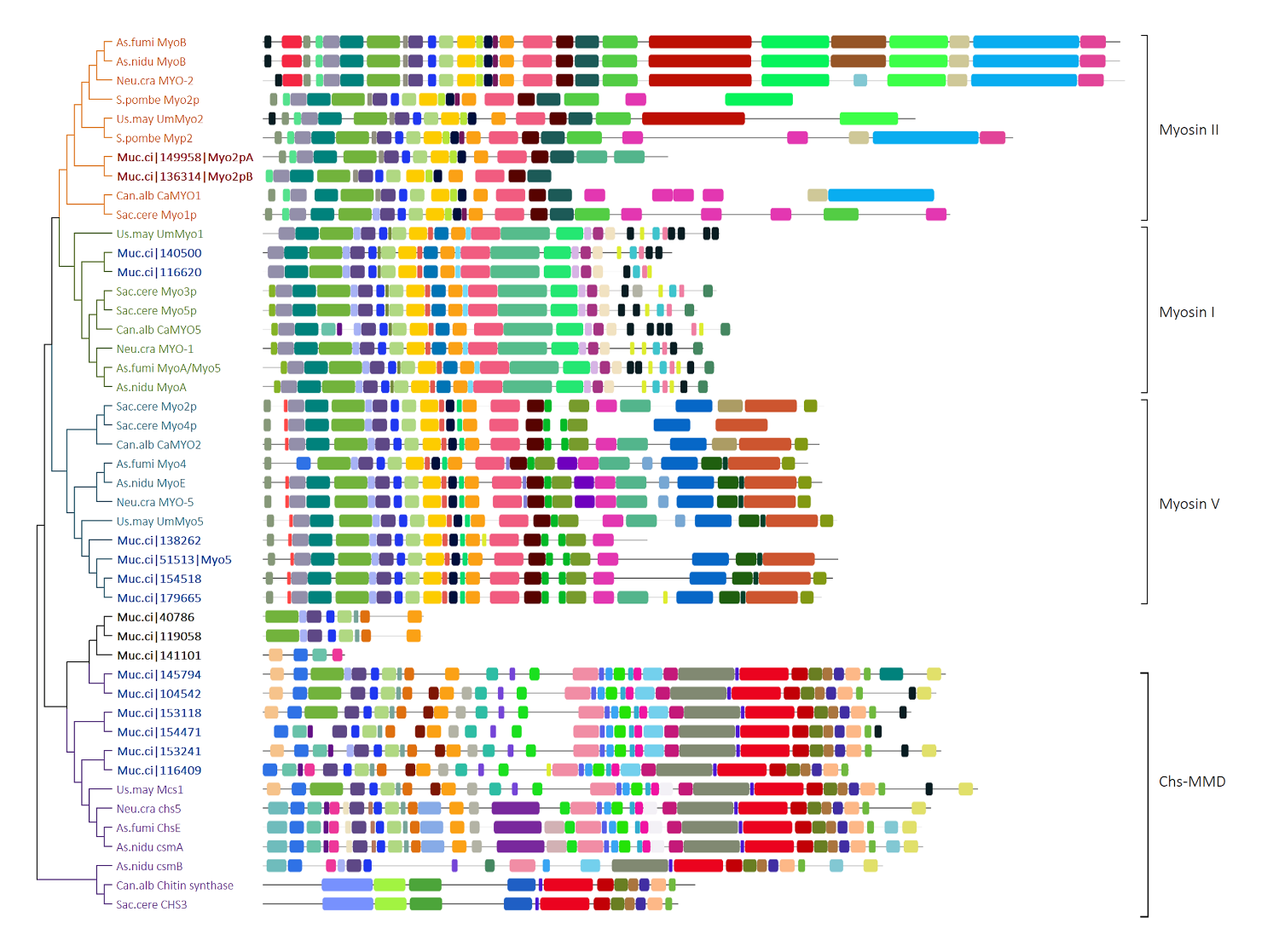


**Supplementary Figure 2.** Phylogenetic and motif discovery analysis of myosin homologs in *Mucor circinelloides* (Muc.ci) and pre-described myosin type II heavy chains in *Aspergillus fumigatus* (As.fumi), *A. nidulans* (As.nidu), *Neurospora crassa* (Neu.cra), *Ustilago maydis* (Us.may), *Candida albicans* (Can.alb), *Saccharomyces cerevisiae* (Sac.cere), and *Schizosaccharomyces pombe* (S. pombe). MEGA X was used to align the amino acid sequences of myosin-II homologs (with the ClustalW algorithm), and to construct a maximum-likelihood (ML) tree.


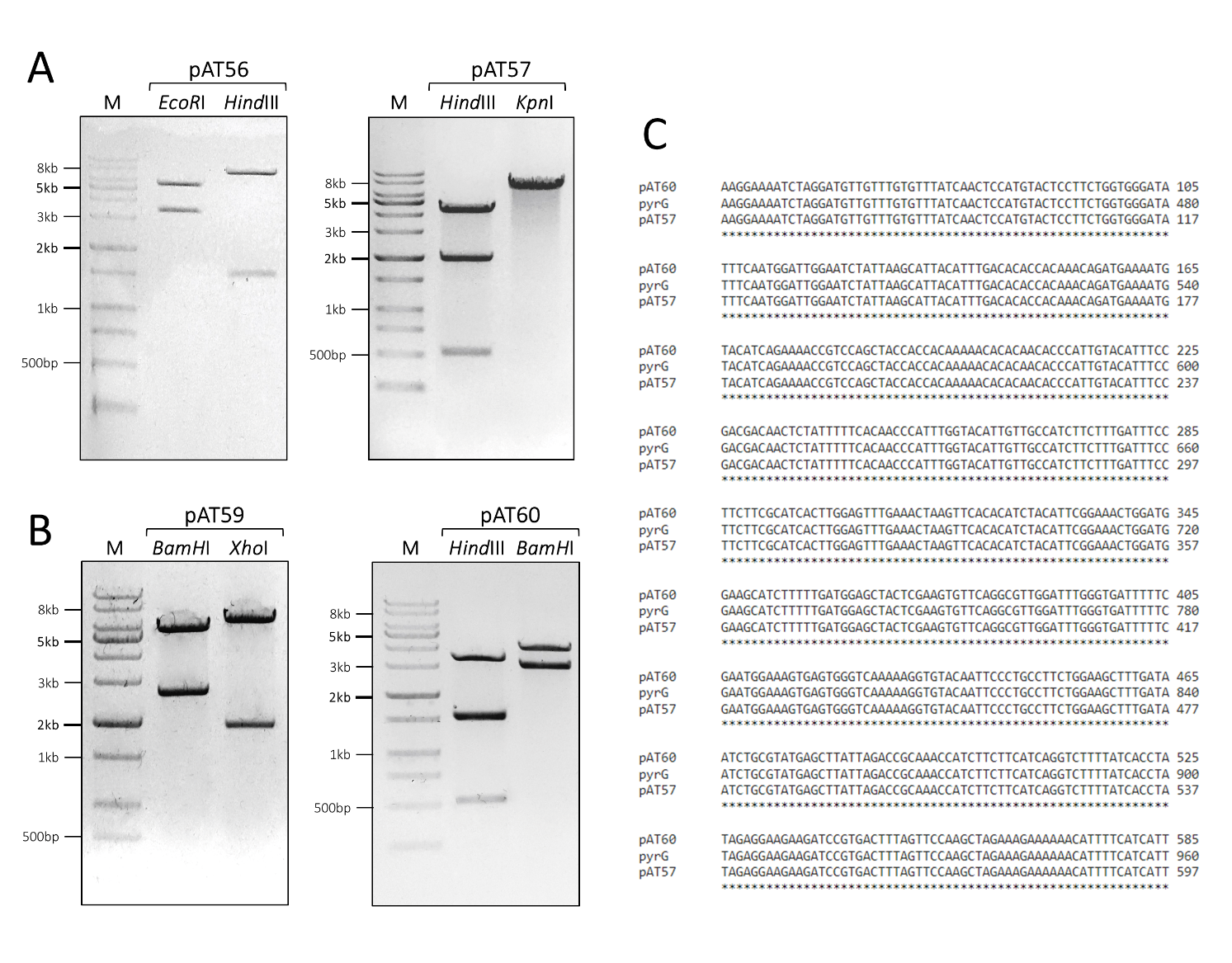
**Supplementary Figure 3.** Verifying recombinant plasmids using restriction enzymes and DNA sequencing**.** The size of DNA fragments resulting from the digestion of pAT56 and pAT57 **(A)** as well as pAT59 and pAT60 **(B)** corresponded with the theoretical calculation. **(C)** Results of DNA sequencing also confirmed that pAT57 and pAT60 contain the sequence of the marker gene *pyrG* flanked between the upstream and downstream regions of the target genes.
